# The Pocket-4-Life project, bioavailability and beneficial properties of the bioactive compounds of espresso coffee and cocoa-based confectionery containing coffee: study protocol for a randomized cross-over trial

**DOI:** 10.1186/s13063-017-2271-2

**Published:** 2017-11-09

**Authors:** Pedro Mena, Michele Tassotti, Daniela Martini, Alice Rosi, Furio Brighenti, Daniele Del Rio

**Affiliations:** Human Nutrition Unit, Department of Food and Drugs, University of Parma, Medical School Building C, Via Volturno, 39, 43125 Parma, Italy

**Keywords:** Coffee, Cocoa, Bioavailability, Pharmacokinetic, Cardiometabolic risk factors, Caffeoylquinic acid, Flavan-3-ols, Caffeine, Trigonelline, Diterpenes

## Abstract

**Background:**

Coffee is an important source of bioactive compounds, including caffeine, phenolic compounds (mainly chlorogenic acids), trigonelline, and diterpenes. Several studies have highlighted the preventive effects of coffee consumption on major cardiometabolic diseases, but the impact of coffee dosage on markers of cardiometabolic risk is not well understood. Moreover, the pool of coffee-derived circulating metabolites and the contribution of each metabolite to disease prevention still need to be evaluated in real-life settings. The aim of this study will be to define the bioavailability and beneficial properties of coffee bioactive compounds on the basis of different levels of consumption, by using an innovative experimental design. The contribution of cocoa-based products containing coffee to the pool of circulating metabolites and their putative bioactivity will also be investigated.

**Methods:**

A three-arm, crossover, randomized trial will be conducted. Twenty-one volunteers will be randomly assigned to consume three treatments in a random order for 1 month: 1 cup of espresso coffee/day, 3 cups of espresso coffee/day, and 1 cup of espresso coffee plus 2 cocoa-based products containing coffee twice per day. The last day of each treatment, blood and urine samples will be collected at specific time points, up to 24 hours following the consumption of the first product. At the end of each treatment the same protocol will be repeated, switching the allocation group. Besides the bioavailability of the coffee/cocoa bioactive compounds, the effect of the coffee/cocoa consumption on several cardiometabolic risk factors (anthropometric measures, blood pressure, inflammatory markers, trimethylamine N-oxide, nitric oxide, blood lipids, fasting indices of glucose/insulin metabolism, DNA damage, eicosanoids, and nutri-metabolomics) will be investigated.

**Discussion:**

Results will provide information on the bioavailability of the main groups of phytochemicals in coffee and on their modulation by the level of consumption. Findings will also show the circulating metabolites and their bioactivity when coffee consumption is substituted with the intake of cocoa-based products containing coffee. Finally, the effect of different levels of 1-month coffee consumption on cardiometabolic risk factors will be elucidated, likely providing additional insights on the role of coffee in the protection against chronic diseases.

**Trial registration:**

ClinicalTrials.gov, NCT03166540. Registered on May 21, 2017.

**Electronic supplementary material:**

The online version of this article (doi:10.1186/s13063-017-2271-2) contains supplementary material, which is available to authorized users.

## Background

Coffee is one of the most popular beverages worldwide and, indeed, its consumption is a moment of pleasure in the daily life of many millions of people. Coffee ranks as the main source of four recognized bioactive constituents within the Mediterranean diet: the purine alkaloid caffeine (1,3,7-trimethylxanthine), the pyridine alkaloid trigonelline, (poly)phenolic substances (mainly chlorogenic acids), and the pentacyclic diterpenes cafestol and kahweol [1]. This unique combination of phytochemicals, with proven biological properties, turns coffee into a dietary agent able to impact on human health [2].

The beneficial properties associated to regular coffee consumption have been clearly described by an important number of systematic reviews and meta-analyses [3–9]. The beverage is associated with a reduced risk of several chronic pathologies related to inflammation processes, such as atherosclerotic heart disease, stroke, and type 2 diabetes, as well as neurodegenerative conditions [4–6, 10–13]. Most of these observations have also emphasized the dose-response inverse relationship of long-term coffee consumption with disease risk. Similarly, the non-linear U-shaped curve linking coffee consumption and cardiovascular disease (CVD) might be due to a combination of beneficial and detrimental effects [4, 14]. However, although observational studies provide the first line of evidence on a causal relation between coffee intake and risk of cardiometabolic diseases, randomized trials are required to address this point definitively [6, 15, 16]. This need for intervention studies has received little attention and, despite some progresses being made [17–19], most of research to date has failed to elucidate the rationale behind the potential preventive effects of coffee consumption. Arguably this can be attributed to the lack of an association of the physiological responses with the coffee bioactives in the circulation. In this sense, coffee is a complex mixture, with dozens of chemicals appearing in the circulatory system after consumption, absorption and metabolism, and where individual circulating metabolites may exert different effects within the human body [20, 21]. The complete pool of coffee-derived circulating metabolites and the contribution of each metabolite to disease prevention are still unknown.

The co-presence in the circulation of different plant-derived metabolites with proven biological activities is a key factor on the prevention of cardiometabolic diseases through adequate dietary habits [1, 22–24]. Among the vegetal matrixes with high content in bioactive phytochemicals, cocoa is gaining increasing attraction. Many initiatives such as the EU FP7 project FLAVIOLA and the COSMOS trial have been carried out in order to assess the efficacy of flavan-3-ols, the main flavonoids in cocoa/chocolate, towards surrogate markers of cardiovascular function, with some of these obtaining positive health claims [25–29]. Cocoa and chocolate also contain high amounts of theobromine (3,7-dimethylxanthine) a closely related analog of caffeine [30]. Cocoa-based products are, therefore, an interesting target that could synergize the preventive cardiometabolic effects of regular coffee consumption. In this sense, chocolate confectionary containing coffee, combining the phytochemical content of coffee and cocoa, could be regarded as a potential candidate to enhance the circulating levels of putatively protective metabolites in the context of a balanced diet.

This work will help to define the bioavailability and beneficial properties of coffee bioactive compounds on the basis of different levels of coffee consumption. Moreover, the contribution of cocoa-based products containing coffee to the pool of circulating metabolites and their putative bioactivity will be taken into account. This innovative study design guarantees adherence to real life settings and patterns of consumption, which will serve to unravel critical gaps within the framework of nutritional intervention studies with coffee.

## Methods/Design

### Objectives

With ultimate aim of studying the bioavailability of coffee/cocoa bioactive compounds and their effects in cardiometabolic health, the objectives of this intervention will be:

(i) ssessing the bioavailability of the four main groups of phytochemicals in roasted coffee (methylxanthines, phenolic compounds, trigonelline, and diterpenes), its modulation by the level of consumption, and establishing the daily average concentration of coffee-derived plasma circulating metabolites;
(ii) investigating the effect of different levels of coffee consumption on cardiometabolic risk factors;
(iii) evaluating circulating metabolites and their putative bioactivity when substituting coffee consumption with the intake of cocoa-based products containing coffee.

### Protocol and study design

A human study will be carried out to achieve the above-described goals. The human intervention study will consist of a short-term, randomized cross-over trial, addressed at measuring the daily mean concentrations of each coffee/cocoa-derived circulating metabolite (CCDCM) for the four main groups of coffee/cocoa phytochemicals (methylxanthines, trigonelline, phenolics, diterpenes). On the basis of different patterns of consumption, this free-living study (although some minimal dietary restrictions will be introduced two days before sampling times) will also take into consideration the effects of repeated doses on the bioavailability of coffee/cocoa bioactives.

**Fig. 1:**
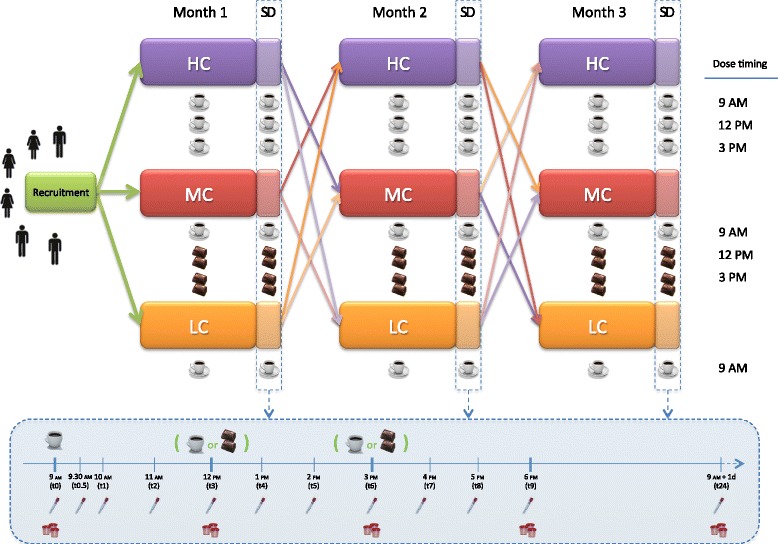
Study design and sampling day (in the blue box) scheme. *HC* high consumers, *LC* low consumers, *MC* medium consumers, *SD* sampling day

**Fig. 2:**
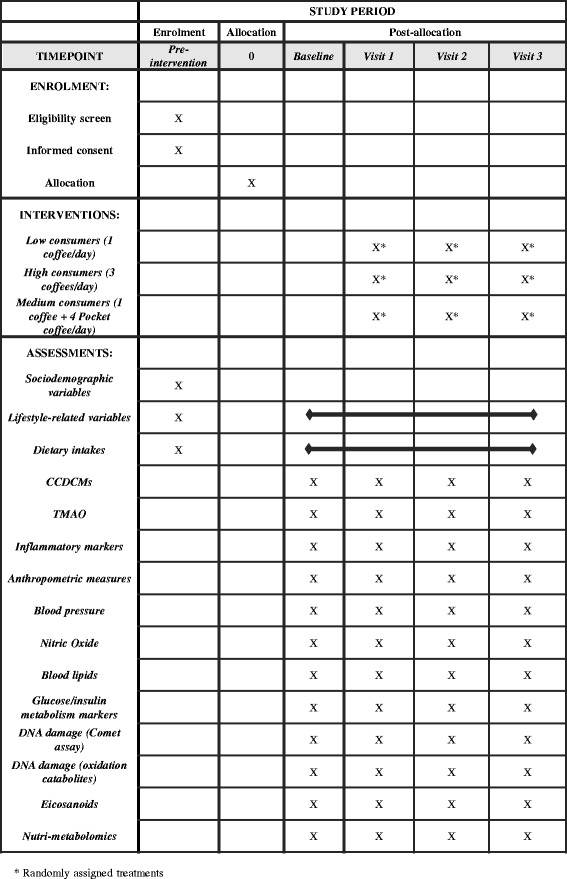
SPIRIT figure: summarizes the allocation, interventions, and outcomes of the study

The study will follow a repeat-dose, 3-arm, cross-over design as shown in Figs. 1 and 2 (SPIRIT chart). This design has been chosen according to ILSI’s guidelines for intervention trials with dietary products [31]. The protocol was also developed in accordance with the Standard Protocol Items: Recommendations for Interventional Trials (SPIRIT) Statement (SPIRIT checklist presented as Additional file 1).

Subjects will be assigned to consume the following treatments in a random order for one month (including the sampling day -the last day of each intervention period-):

1. one cup of espresso coffee/day (“low consumers”) at 9.00 AM;
2. three cups of espresso coffee/day (“high consumers”) at 9.00 AM, 12.00 PM, and 3.00 PM;
3. one cup of espresso coffee at 9.00 AM and two cocoa-based products containing coffee two times per day, at 12.00 PM and 3.00 PM. This group will be named “medium consumers”, in view of the caffeine content of the cocoa-based product containing coffee.

Minimal recommendations to avoid other sources of coffee/cocoa-related phytochemicals besides those introduced through the assigned treatment, and to standardize the time of coffee consumption, will be provided for the 2 days prior to each sampling day and on the sampling day. Dinner timing and composition will also be standardized the day before the sampling day. Only water could be drunk during the night. In the morning of the sampling day, the subjects will attend a clinic where fasting baseline blood and urine samples will be collected. Then, low and high consumers will drink one or three cups of espresso coffee, respectively (without sugar, sweeteners, and milk for the first coffee; with 5 g of sugar for the last two coffees), while medium consumers will drink a cup of espresso coffee and two cocoa-based products containing coffee twice during the course of the day, following the above mentioned timing. After ingestion of the first coffee together with a polyphenol-free breakfast (a sponge milky cake), blood and urine samples will be collected at selected time points over the ensuing 24-h time points (Fig. 1). Five hours after the consumption of the first coffee, participants will receive a standardized mixed meal (ham and cheese sandwich) free of coffee/cocoa-related phytochemicals. Water will be available ad libitum. Twenty-four hours after receiving the treatment, blood and urine samples will also be taken in order to assess return to baseline. In addition, anthropometric characteristics and blood pressure will be measured.

### Testing materials and phytochemical composition

Volunteers will be supplied with a single-serve coffee machine (Essenza EN 97.W, De’Longhi Appliances S.r.l, Treviso, Italy) and coffee capsules (Capriccio, Nespresso Italia S.p.a., Assago, Italy) to standardize brewing method, raw material, and cup volume (approximately 45 mL). Volunteers will also be supplied with the cocoa-based product containing coffee (Pocket Coffee, Ferrero Commerciale Italia S.r.l., Alba, Italy). The content in phytochemicals of the coffee and the cocoa-based product containing coffee will be analyzed by ultra-performance liquid chromatography-tandem mass spectrometry (UHPLC-MS/MS) following methodologies described in previous reports [32–35]. Accurate calibration and absolute quantification will be achieved by comparison with authentic standards. The amount of caffeine provided by a cup of espresso coffee is approximately 60 mg/serving, while two cocoa-based products containing coffee provide approximately 30 mg of caffeine.

### Participant selection

Twenty-one healthy volunteers will be recruited in Parma (North Italy) for the study, using announcements placed in university, hospital, and public places. All subjects potentially involved in the nutritional intervention will be informed of the details of the protocol and about the risks associated with participation. Those who agree to participate will be asked by the study staff for their written informed consent to participate in the study. Information to the volunteers will be provided before and separately from the consent form. Personal data collection will include names, e-mail, phone number, age, height, weight, and dietary habits of the volunteers.

### Inclusion and exclusion criteria

Inclusion criteria will include being adult, healthy, of normal weight [body mass index (BMI) 18–25], and regular consumers of one to five cups of coffee per day. Exclusion criteria will include clinical diagnosis for metabolic, renal or digestive disorders, regular consumption of medication, antibiotic therapy taken within the last 3 months, intense physical activity, pregnancy or lactation, regular intake of coffee exceeding five coffees per day, and very high consumption of coffee/cocoa-related phytochemicals. These criteria are set in order to avoid likely confounding factors [36].

### Data and sample collection

Socio-demographic variables will be assessed through a generic questionnaire completed at recruitment. The questionnaire will also contain questions useful to identify possible exclusion criteria (e.g. diagnosis for diseases, regular consumption of medication, food allergy). Dietary habits of volunteers will also be evaluated during the enrollment, through a semi-quantitative food frequency questionnaire (FFQ) for the assessment of dietary total antioxidant capacity [37]. In addition, volunteer’s food intake and compliance with the study requirements will be assessed by means of 3-day dietary records, administered throughout each intervention period at two time points: (i) in the middle of each intervention period during 2 weekdays and a weekend day, and (ii) at the end of each intervention period, 2 days prior to the sampling day and the sampling day. The habitual physical activity level of each participant will be measured through a validated International Physical Activity Questionnaires [38].

Blood sample collection will be carried out in the clinic unit of the Department of Medicine and Surgery. Blood collection will be carried out by a physician. A venous catheter will be inserted into the antecubital vein and blood samples from each subject will be collected in specific tubes at the time points indicated at Fig. 1. Blood at 24 h after first coffee consumption will be taken by venipuncture. Urine samples will be collected during different periods of time (Fig. 1) using urine collectors.

Blood samples will be centrifuged and plasma, serum, and peripheral blood mononuclear cells (PBMCs) will be collected, aliquoted, and stored at -80 °C for further processing. The volume of urine excreted will be measured and aliquots stored at -80 °C for further processing.

### Measurements

The primary selected endpoint of the study is the quantification of the daily mean concentration of coffee-derived plasma circulating phenolic metabolites, whereas the study of the bioavailability of other coffee-derived circulating bioactives, the bioavailability of cocoa-derived circulating phytochemicals, and the assessment of cardiometabolic markers will be considered secondary endpoints.

### Anthropometric measures

At the beginning and end of each intervention period, body weight, height, and waist circumference will be assessed, and BMI will be calculated.

### Blood pressure

Systolic and diastolic blood pressure of each volunteer will be obtained after a 5-min rest in a seated position in the morning of the beginning and end of each intervention period.

### Daily mean concentration of coffee/cocoa-derived plasma circulating phenolic metabolites

The determination of CCDCMs will be performed in all the samples. Samples will be subjected to UHPLC-MS^n^ analysis (linear ion trap MS for identification and triple quadrupole MS for quantification purposes). Plasma samples will be extracted according to Zhang et al. [39], while urine samples will be centrifuged, diluted, and filtered. Methylxanthines, trigonelline, other pyridine metabolites, diterpenes, and phenolic metabolites will be determined as previously described [32–35]. Accurate calibration and absolute quantification will be achieved by comparison with authentic standards when commercially available or with standards previously synthesized [40, 41].

### Pharmacokinetic studies

**Fig. 3:**
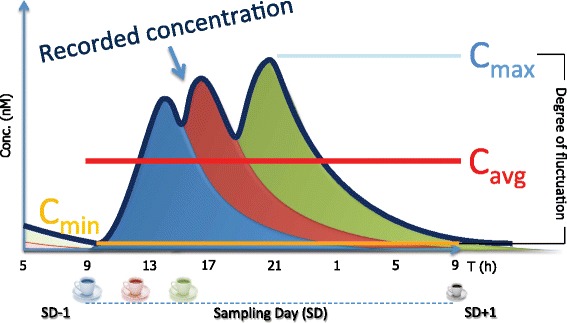
Modelled pharmacokinetic profile recorded after repeated consumption of coffee along the day. *C*_*max*_ maximum concentration, *C*_*avg*_ average concentration, *C*_*min*_ minimum concentration, *SD* sampling day

Metabolite data will be analyzed using the WinNonlin software (Certara, LP, Princeton, NJ, USA). Pharmacokinetic parameters of each volunteer will include maximum, minimum, and average plasma concentration (C_max_, C_min_, and C_avg_), degree of fluctuation, area under the curve from 0–24 hours (AUC_0–24_), times of maximum and minimum plasma concentrations (T_max_ and T_min_), and they will be calculated for each metabolite (Fig. 3). Urinary excretion kinetics will also be estimated.

### Trimethylamine N-oxide

Trimethylamine N-oxide (TMAO) is a novel biomarker of cardiovascular risk produced from L-carnitine with involvement of the gut microbiota and, thus, influenced by the diet [42, 43]. It will be quantified by UHPLC-MS^n^ in baseline (0 h) plasma samples before and after each treatment. Before the analysis, TMAO-d9 will be added to samples as internal standard after which the plasma will be extracted with acidified acetonitrile as described previously [44]. Samples will be centrifuged and the supernatants collected for the UHPLC-MS^n^ analysis.

### Inflammatory markers

Some markers associated with inflammatory processes linked to the onset of atherosclerosis will be analyzed with a Bio-Plex Pro™ Human Cytokine Assays (Bio-Rad Laboratories S.r.l., Segrate, Italy). The concentrations of interferon gamma (IFN-γ), interleukin (IL)-1β, IL-2, IL-4, IL-5, IL-6, IL-7, IL-8, IL-10, IL-12 (p70), IL-13, IL-17, monocyte chemotactic protein 1 (MCP-1), and tumor necrosis factor alpha (TNF-α) will be measured by using a Bio-Plex® MAGPIX™ Multiplex Reader (Bio-Rad Laboratories). These analyses will be performed in the baseline (0 h) plasma before and after each treatment.

### Nitric oxide

Increased nitric oxide (NO) bioavailability is inversely associated with endothelial dysfunction. NO acts as an endogenous vasodilatory factor involved in the regulation of the endothelial function, maintaining an anti-inflammatory environment in the vessel [45]. Plasmatic NO, assessed as nitrate/nitrite, will be determined at the beginning and end of each intervention period using Nitrate/Nitrite Fluorometric Assay Kit (Vinci-Biochem S.r.l., Vinci, Italy).

### Blood lipids

Plasma total cholesterol, high-density lipoprotein (HDL)-cholesterol, low-density lipoprotein (LDL)-cholesterol, and triglycerides will be analyzed by standardized routine hospital protocols at the beginning and end of each intervention period.

### Fasting indices of glucose/insulin metabolism

In order to investigate the effect of the chronic coffee consumption on diabetes and insulin resistance prevention, plasma glucose and insulin concentrations will be monitored at fasting state before and after each intervention period. The analysis of glycemia will be performed by means of an automatic analyzer with a combined enzymatic-electrochemical system (YSI 2900 STAT PLUS, Yellow Springs Instruments, Yellow Springs, OH, USA). Plasma insulin concentration will be monitored by using routine blood analysis. Fasting insulin sensitivity will be determined using the quantitative insulin sensitivity check index (QUICKI) [46]. Fasting insulin secretion capacity will be evaluated as the homeostatic model assessment (HOMA) for beta cell function (HOMA-B) [47] and for insulin resistance (HOMA-IR) [48].

### DNA damage (Comet assay)

H_2_O_2_-induced DNA damage (i.e. oxidatively induced DNA damage) and formamidopyrimidine DNA glycosylase-sensitive sites of DNA (i.e. endogenous oxidative base damage) will be evaluated by the Comet assay, a procedure for the evaluation of DNA damage in all types of eukaryotic cells and tissues. DNA damage will be evaluated in PBMCs before and after each treatment, in parallel with the pharmacokinetic study in order to determine the effect of both acute and long-term consumption [49].

### DNA damage (oxidation catabolites)

DNA oxidation catabolites (8-hydroxy-2′-deoxyguanosine, 8-hydroxyguanosine, 8-nitroguanosine, guanosine-3′-5′-cyclic monophosphate, and 8-nitroguanosine-3′-5′-cyclic monophosphate) will be measured in plasma, collected before and after each treatment, by UHPLC-MS/MS, in parallel with the pharmacokinetic study in order to determine the effect of both acute and long-term consumption [50].

### Eicosanoids (oxidative stress, vascular, and inflammatory markers)

Eicosanoids are biomarkers for tracking changes in lipid peroxidation and vascular events. The eicosanoid family comprises prostaglandins, thromboxanes, and isoprostanes. These markers will be evaluated in urine before and after each treatment by UHPLC-MS/MS [51].

### Nutri-metabolomics

Urine and plasma samples at the beginning and end of each intervention period will be subjected to untargeted high-resolution-liquid chromatography mass spectrometry (HR-LC-MS/MS) metabolomics and lipidomics approaches, in order to assess potential differences between control (before) and treatment (after), as well as among the three treatments described. Metabolomics analysis and data processing will be carried out following previous works in the field [52, 53], as well as lipidomics [54]. Both works will be carried out by using a Vion Ion Mobility Quadrupole Time of Flight Mass Spectrometry (Waters, Milford, MA, USA). This explorative investigation will be applied as a complementary assay to generate a comprehensive picture of the impact of coffee and the cocoa-based product containing coffee on human health.

### Sample size calculation, randomization and statistical analysis

The sample size has been calculated considering the primary outcome, the daily mean concentration of coffee-derived plasma circulating phenolic metabolites, and according to Lenth [55]. Considering the lack of literature dealing with this outcome, the AUC of dihydrocaffeic acid-3′-sulfate, one of the most representative coffee-derived phenolic metabolites [56], has been used for sample size calculations. Keeping a 80% power and an α of 5%, and considering data from Stalmach et al. [56], 15 subjects will have to complete an acute intervention to detect a change of 600 nmol/h/L^-1^ in dihydrocaffeic acid-3′-sulfate plasma concentration with a standard deviation of 870 nmol/h/L^-1^. A total of 21 subjects will be recruited to allow for dropouts and for nonparametric statistical analysis (15% additional subjects required). Power calculation will be retrospectively calculated for the secondary cardiometabolic outcomes (post hoc sample size calculation). Once the volunteers have been recruited, a randomization list will be generated using a randomized block design by means of Random Number Generator Pro (Segobit Software). This list will be made by a colleague not involved in subject enrollment, and using a numbered sequence in sealed, opaque envelopes. It will be blind for the Principal Investigator (PI) and volunteers.

Before any comparison is performed, the normality of each variable will be evaluated to choose the most appropriate statistical test. If normally distributed, data will be expressed as mean ± standard deviation and analyzed using general linear models for repeated measures with post hoc comparisons. If data are not normally distributed, they will be reported as median and interquartile range and the Friedman test with post hoc pairwise comparisons will be performed. Multivariate analyses will be carried out to understand individual responses to coffee consumption on the basis of metabolite production. All the analyses will be carried out using SPSS 23.0 (IBM Corp., Armonk, NY, USA). *P* value <0.05 will be regarded as statistically significant.

### Confidentiality of data

Each participant will be assigned to a unique code provided by the PI, so that all personal information, including questionnaire information and samples, will be confidential. Information will be collected exclusively by the PI (or the specialized staff officially involved in the study) and will be stored in a dedicated non-web-connected computer. Sample codification will be hidden to the researchers analyzing the samples (single-blind study). Recruited volunteers will sign an authorization to the use of personal information and data. The identity of the participants will not be revealed in any published data or in presentation of the information obtained as a result of this study. All the data collected for this study will be treated as confidential. All biological specimens will be destroyed after the analysis, as indicated in the informed consent given to the volunteers and according to the procedures of the University of Parma.

## Discussion

There is growing evidence that regular coffee consumption is associated to several beneficial effects on health. However, research has emphasized how differences in the number of cups consumed on a daily basis affect the prevalence of cardiometabolic disorders [15, 57–63] and biomarkers of cardiometabolic risk [15, 61, 64]. There is a linear inverse dose-response relationship between coffee consumption and diabetes, and every additional cup of coffee in a day is associated with a 5% to 10% lower risk of new-onset disease [6, 60, 65]. In the case of CVD, a non-linear U-shaped curve links coffee consumption and CVD risk, with the largest risk reduction observed at a level of about three cups/day, although high rates of variability have been observed [59, 66, 67]. These difficulties in defining the required coffee intake to promote cardiometabolic health make dietary recommendations for coffee consumption almost impossible [12]. Thus, randomized trials addressing the impact of coffee dose on markers of cardiometabolic risk are needed [6, 15, 16].

Intervention studies considering coffee dosage are scarce, and the biological actions underlying the preventive effects of coffee consumption has not been elucidated. The main reasons behind this limited information may be (1) the lack studies considering the association of the physiological responses with coffee bioactives in circulation, and (2) the high inter-individual variation observed for the selected cardiometabolic endpoints. Regarding point 1, it should be noted that ascertaining the exact metabolites appearing in circulation after consuming a cup of coffee is a key point to fully unravel the bioactive(s) responsible for its preventive effects. Nevertheless, despite substantial research on caffeine, trigonelline, and phenolic compounds [33], there is a lack of fundamental knowledge on this critical topic, even more when it comes to different patterns of consumption including the number of daily servings and repeated daily doses, which represents a common scenario among coffee consumers. In addition, the circulating metabolites derived from coffee diterpenes [35, 68, 69], potent cholesterol-raising compounds, are still unknown. This information is paramount to draw a more realistic physiological picture and, hence, to better understand the biological properties of long-term coffee consumption [33, 61]. On the other hand, heterogeneity in individual responsiveness to food components (point 2) can obscure associations between diet and health, and limit the understanding of the exact role of the different coffee bioactives. Individual responses to coffee consumption, potentially driven by variations in the bioavailability of key metabolites [70], may be affected by gender [18, 60], age [71], weight or BMI [18, 60], health status [15], genetic polymorphisms [72, 73], smoking [60], physical activity [74], dietary habits, and gut microbiota composition [75–78], among other factors. The gut microbiota has recently emerged as one of the key drivers for diet: cardiometabolic health interactions, due to its ability to produce several metabolites that modulate host physiology at many levels [77, 79, 80]. Likewise, coffee phenolics are highly metabolized by the gut microbiota and are able, in turn, to modulate the microbiota composition [78, 81–83]. This bidirectional relation may deeply condition both the cardiometabolic response and the types and levels of circulating bioactives after coffee consumption.

Attending to the real-life approach of this intervention study, the presence in volunteers’ diets of other sources of phytochemicals cannot be avoided. The intake of amounts of methylxanthines, (poly)phenolic compounds, and trigonelline not provided in the framework of the intervention may thus represent a potential confounding factor for the cardiometabolic outcomes. Three considerations are taken into account to control these variables. First, subjects consuming high amounts of coffee/cocoa bioactives, as assessed by the FFQ provided during the recruitment, will not be enrolled. Second, dietary information collected from the 3-day dietary records, administered in the middle of each intervention period, will be used to verify the information provided by the FFQ. If significant differences are observed among volunteers, the intake of phenolics, methylxanthines, and trigonelline, as assessed by in-house databases, could be used as covariates. Lastly, volunteers will be stratified according to their dietary total antioxidant capacity (TAC) [37] and results for cardiometabolic outcomes will be provided for two separate groups (low and high TAC).

In summary, despite an overwhelming number of published studies, there is a paradoxical lack of knowledge on the bioactives responsible for the observed beneficial effects of coffee, and on their ability to regulate physiological processes involved in its preventive effects. This missing information becomes even more apparent when the effect of dosage and repeated doses during the day are taken into account. Finally, although some factors influencing inter-individual variability have been tackled, most remain still unexplored. This study will try to bridge these major gaps by linking coffee consumption to individual CCDCM profiles and metabolic responses. To do that, in a real-life setting, the design of this study is extremely innovative, joining under the same protocol both acute and short-term observations, maximizing its prospects and reducing all the operational constraints associated with separate intervention studies.

### Trial status

This study is currently recruiting participants. The recruitment started at the beginning of May 2017 and is expected to end by the end of June 2017.

## Additional file

Additional file 1: SPIRIT 2013 checklist: recommended items to address in a clinical trial protocol and related documents. (DOC 121 kb)

## Abbreviations

AUC: Area under the curve
BMI: Body mass index
CCDCMs: Coffee/cocoa-derived circulating metabolites
CVD: Cardiovascular disease
FFQ: Food frequency questionnaire
HOMA: Homeostatic model assessment
HR-LC-MS/MS: High-resolution-liquid chromatography mass spectrometry
IL: Interleukin
NO: Nitric oxide
PBMCs: Peripheral blood mononuclear cells
PI: Principal Investigator
TAC: Total antioxidant capacity
TMAO: Trimethylamine N-oxide
UHPLC-MS^n^: Ultra-high pressure liquid chromatography-mass spectrometry
